# Endoscopic surgery of an extensive aneurysmatic bone cyst of the paranasal sinuses in a 12-year-old patient^☆^^☆☆^

**DOI:** 10.1016/j.bjorl.2016.04.008

**Published:** 2016-05-06

**Authors:** Axel Wolf, Wolfgang Koele, Manfred Ratschek, Doris Lang-Loidolt, Peter Valentin Tomazic

**Affiliations:** a Medical University of Graz, Department of Otorhinolaryngology, Graz, Austria; b Medical University of Graz, Institute for Pathology, Graz, Austria

## Introduction

Aneurysmatic bone cysts (ABCs) are rare, non-neoplastic lesions firstly described by Lichtenstein in 1945. These bony-cystic lesions are frequently filled with blood and destroy the architecture of the affected bone and surrounding tissue.^1^, ^2^ Although histopathological and radiologic characteristics of ABCs are well defined, pathogenesis is not clearly investigated yet: most probably local blood circulation disturbance can cause ABCs.^3^

Usually metaphysis of long bones and the vertebral column are affect by ABCs. Only about 2% of ABCs occur in the head and neck region, mainly affecting the mandible.^1^, ^3^, ^4^

Other benign bony lesions such as non-ossifying fibroma, giant cell granuloma, fibrous dysplasia and fibromyxoma can be associated with these pseudocysts.^4^ Although radical surgery is the gold-standard therapy for ABCs, this cannot be achieved in all cases due to the occurrence of extensive tumors at difficult anatomical structures.^2^ Moreover in benign lesions surgical mutilation particularly in young patients should be avoided.

Due to the rarity of ABCs in paranasal sinuses and the emerging role of endonasal endoscopic sinus surgery (ESS) over the last decades we want to report about an extensive lesion treated with ESS.

## Case report

A 12-year-old girl was referred to our department because of persisting nasal obstruction and occasional epistaxis.

**Figure 1:**
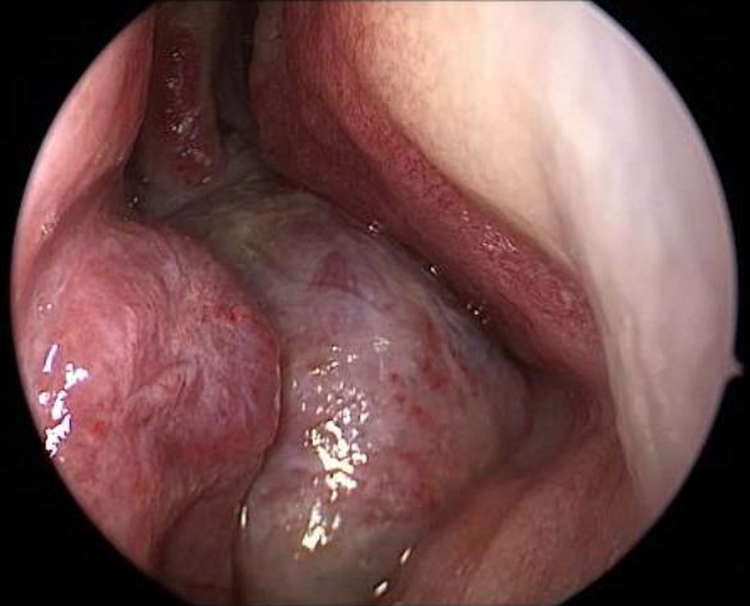
Endonasal endoscopy of the right nasal cavity: appearance of the tumor mass.

Endoscopy showed a cystic tumor like mass (Fig. 1) obstructing the right nasal cavity. Furthermore a septal deviation to the left side with total obliteration of the left nasal cavity was diagnosed. General clinical examinations and routine blood analysis revealed normal health condition of the patient. There was no history of trauma and no obvious impairment of ocular movement, vision, pupillary reaction or proptosis was observed.

**Figure 2:**
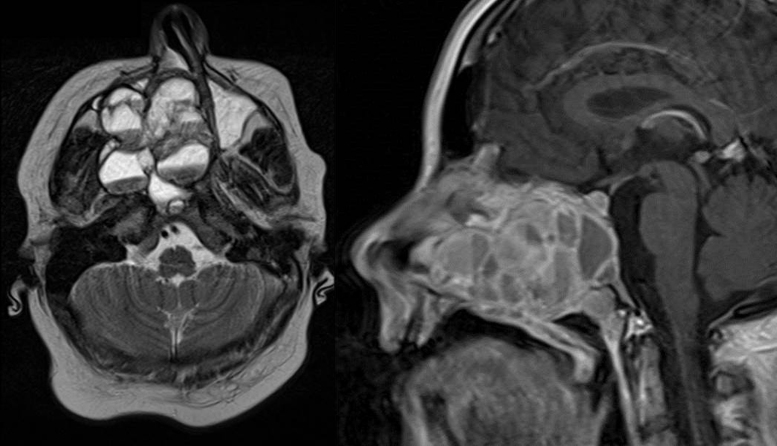
MRI of the head. Axial T1 weighted image (left) and sagittal T1 weighted image (right): an extensive iso- to hypertense lesion of the midface with multilocular conspicuous fluid levels, enhancing septations and solid components. Tumor affected the medial, lateral and ventral wall of the right maxillary sinus. Contralateral it destructed the medial wall of the left maxillary sinus. Erosions of the right bony palate and of major parts of the dorsal septum and the inferior concha nasalis on both sides were observed. The dorsal expansion leads to the clivius, which was partly destructed, and to destruction of the pterygoideus process on the right. Radiologic investigations furthermore revealed partly destruction of the medial wall of the internal carotic artery and partly destruction of the orbital floor and lamina papyracea right.

Magnetic Resonance Imaging (MRI) as well as Computed Tomography (CT) for computer assisted navigation was performed showing a 6.6 × 5.1 × 5 cm large expansion in the nasal cavity reaching skull base (Fig. 2). The right orbital wall as well as the internal carotid artery canal appeared compressed. On MRI the lesion showed multiple cysts filled with liquid. A transnasal endoscopic biopsy and subsequent excision with computer assisted navigation control was performed after having excluded a juvenile nasal angiofibroma by means of angiography.

**Figure 3:**
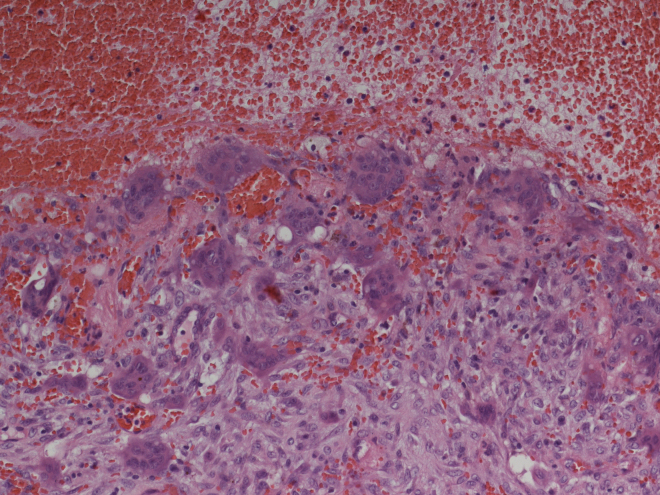
Histopathologic examination: aneurysmatic bone cyst. Microscopy showed multiple cystic spaces filled with blood separated by septae. Fibroblasts, chronic inflammatory cells, giant cells and osteoid without cell atypies were observed. In synopsis with radiologic results and blood tests giant cell tumors, giant cell granuloma and a giant cell tumor in association with hyperparathyroidism were be excluded.

Preoperative examinations revealed that radical tumor resection would not be achieved, thus, the lesion was maximally debulked under endoscopically with minimal blood loss. Histologic examination revealed an ABC (Fig. 3).

**Figure 4:**
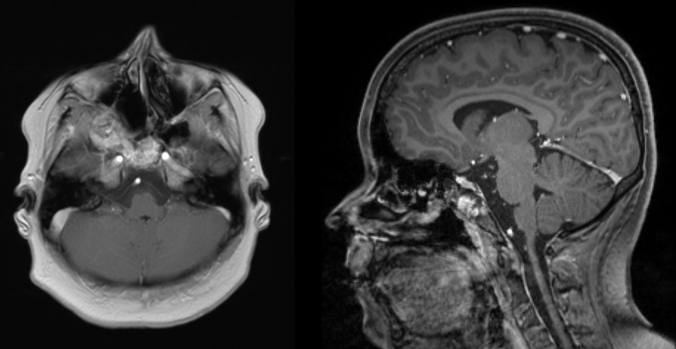
MRI of the head 18 months after primary surgery. Axial T1 weighted image (left) and sagittal T1 weighted image (right): tumor mass was significantly reduced after the first intervention. A minimal residual tumor affecting the clivus and the ethmoid bone can be observed but lesions do not cause any clinical symptoms.

As expected postoperative MRI showed a residual tumor in the area of the clivus and ethmoidal bone (Fig. 4), nevertheless the girl was free from symptoms and without endoscopic signs of progression two years postoperatively given an optimal cosmetic outcome due to the approach.

## Discussion

ABCs are benign cystic lesions that destroy and expand the affected bone.^1^, ^2^, ^4^ Radiologic appearance similar to vascular aneurysms lead to the name ‘ABC’.^2^ Primary and secondary lesions can be differentiated: primary ABCs have no history of trauma or other associated tumors, while one third of the lesions occur secondary to other tumors such as giant cell tumor or chondroblastoma.^2^, ^5^ Due to the uneventful medical history of our young patient the lesion can be defined as ‘primary’ ABC. No difference in distribution of sex of the disease is described.^5^ Typically ABCs occur in thorax, pelvis and long bone metaphysis and only two percent of diseases occur in the head and neck region. Furthermore, most cases in the head and neck region affect the mandible.^2^ After literature review we could find about 24 cases affecting paranasal sinuses none of which was as extensive as in the present case (Table 1).

**Table 1:** Information about cases of ABCs of the paranasal sinuses published previously.

Author	Year	Age	Location	Symptoms	Treatment	Approach	Resection	Follow-up	Recurrence
Kimmelman et al.	1982	10	Sphenoid sinus	Visual loss	Resection	Combined	R0	6 Years	No
Yee et al.	1977	10	Sphenoid and ethmoid sinus	Visual loss	Resection	External	R+	1 Year	
Som et al.	1991	16	Sphenoid and ethmoid sinus	Anosmia, headache	Resection	Undefined	Undefined	Undefined	Undefined
Saito et al.	1998	11	Sphenoid sinus	Nasal obstruction	Resection	Undefined	Undefined	3 years	Undefined
de Minteguiga et al.	2001	14	Sphenoid and ethmoid sinus	Headache	Embolization and resection	Internal	R0	3 Years	No
Cansiz et al.	2002	17	Sphenoid sinus	Headache	Resection	External	R0	3 Years	Undefined
Gan et al.	2001	28	Sphenoid sinus	Headache, diplopia	Resection	Internal	R+	3 Years	
Hunter et al.	1990	7	Sphenoid sinus and orbit	Periorbital swelling	Resection	External	R0	1 Year	No
Chartrand-Lefebvre et al.	1996	4	Sphenoid sinus	Ptosis, strabisumus	Embolization, resection and sclerotherapy (2 stage intervention)	External	R0	2 Years	
Tamini et al.	2005	14	Sphenoid sinus, orbit and skull base	Headache, visual loss	Resection	External	R0	Undefined	No
Gan et al.	2007	5	Sphenoid sinus, orbit and temporal fossa	Diplopia	Resection	Undefined	Undefined	3 Years	Undefined
Cocukta et al.	2009	9	Sphenoid sinus, ethmoid sinus and orbit	Nasal obstruction, headache	Resection	External	R0	2 Years	No
Fikri et al.	2013	12	Ethmoid sinus	Not defined	Embolization and resection	Undefined	Undefined	Undefined	Undefined
Nadkarni and al.	2001	19	Sphenoid and ethmoid sinus and orbit	Nasal obstruction, visual loss, epistaxis, ansomia	Resection	External	R0	1 year	No
Yee et al.	1977	10	Sphenoid and ethmoid sinus and orbit	Visual loss	Resection	External	R1	1 Year	
Goyal et al.	2012	14	Sphenoid and ethmoid sinus	Nasal obstruction, cheek swelling	Resection	External	R1	3 Months	
Terkawi et al.	2005	7	Sphenoid sinus, ethmoid sinus and orbit	Nasal obstruction, visual loss, proptosis	Resection	Combined	R1	5 Months	1
Hnenny et al.	2015	28	Sphenoid sinus, maxillary sinus, ethnoid sinus, orbit and skull base	Ptosis, diplopia	Resection + embolization	External	R0	3 Months	No
Hashemi et al.	2015	5	Sphenoid sinus	Ptosis	Resection	Endoscopic	R1	6 Months	
Tang et al.	2009	17	Maxillary sinus	Cheek swelling, nasal obstruction	Resection + embolization	External	R0	4 Years	No
Bozbuga et al.	2009	9	Sphenoid sinus, orbit and skull base	Nasal obstruction, headache	Resection + embolization	External	R0	22 Months	No
Janjua et al.	2014	30	Ethmoid sinus	Headache, diplopia	No surgery	No intervention		4 Years	
Verma et al.	2013	8	Maxillary sinus	Extopic molar tooth	Resection	Internal	R0	1 Year	No
Salmasi et al.	2011	16	Sphenoid sinus	Visual loss	Resection	Internal	R0	6 Months	No
Sinha et al.	2010	13	Ethmoid bone and orbit	Visual loss	Resection	Undefined	Undefined	Undefined	Undefined

Symptoms are mostly described with headache, ptosis, rhinorrhoea, strabismus, exophthalmos, swelling, vision loss and nasal obstruction.^1^, ^2^ The girl in our report suffered from nasal obstruction and recurrent epistaxis only although the massive extension and location of the lesion. As the patients was firstly presented with suspect hypertrophic adenoids to an otorhinolaryngologist, this case shows again that endonasal endoscopy is an essential examination to exclude other, less common causes for nasal obstruction.

Age of diagnosis of ABCs of the paranasal sinuses in previous cases was mainly below 20 years but also cases of considerably older patients were reported.^2^ Our 12-year-old patient was in the typical age group at the time of primary diagnosis.

After clinical examination, radiologic investigations must be performed for further diagnosis. In CT scans these lesions show a typical multicystic appearance with bony architecture and well demarked margins. Cysts are often filled with blood clots. In T2 weighted imaging ABCs show intracystic components of heterogenic enhanced signals with fluid levels and peripheral septae that appear isodense with marked enhancement.^6^, ^7^ As shown in Figure 1, Figure 2 imaging of our young patient showed characteristic signs of ABC. Although radiologic finding in these lesions are well defined, MRI and CT scans are characteristic but not specific for diagnosis.^1^ Histopathological investigation is essential for further diagnosis. CT-angiography should be performed before biopsy in order to exclude high vascularized tumors, e.g. angiofibroma to avoid complications such as heavy bleeding.

In histological examination malignancy of the tumor must be ruled out and potential underlying precursor lesions such as e.g. osteoblastoma, chondroblastoma, giant cell tumors, etc. should be defined or excluded; histological appearances of ABCs in the skull are the same is in the long bones. Blood filled pseudocysts surrounded by fibrous tissue in which giant cells are commonly find are typical for these bony lesions. They are defined as pseudocysts due to the missing epithelial layer on the surface of the cystic formations.^1^, ^4^, ^7^, ^8^

As performed in our case, definitive diagnosis of ABCs can only be obtained in synopsis of interviews, clinical examination, imaging and histopathologic examination.

Gold-standard treatment for ABCs is the total resection of the lesion if possible.^2^, ^4^, ^5^

Adjuvant arterial embolization to avoid intraoperative bleeding is described previous reports (Table 1). In the present case only minimal blood loss occurred during surgery, thus, we would not generally recommend preoperative arterial embolization. Other treatment options e.g. tumor enucleation or curettage can be applied in extensive, unresectable lesion but are usually not used in diseases of paranasal sinuses and high recurrence rates from about 26–60% are reported in literature.^5^, ^9^ Radiotherapy is generally not used for treatment of ABCs but can be part of the therapeutic scheme in unresectable, recurrent lesions.^5^ Furthermore, sclerotherapy and cryosurgery may be an alternative in unresectable, recurrent tumors although, again, experience of its application in ABCs is very limited.^7^, ^10^

For tumor resection different surgical approaches according to lesion size and location can be used.^11^ Lesions can be resected using external approaches, e.g. lateral rhinotomy or ESS. Due to the size and localization of the ABC no total tumor resection was possible in this case, thus, we decided to use the minimal invasive, endoscopic surgery for tumor debulking in order to achieve an improvement of symptoms for the patient with optimal cosmetic outcome.

The endoscopic approach is less invasive, leads to faster recovery of patients and to a better postoperative cosmetic outcome particularly important in young patients. Major parts of the tumor were resected endoscopically.

Two years after the surgery our patient was totally free of symptoms. In nasal endoscopy no tumor progression was observed although MRI still showed a minimal residual tumor in the area of the clivus and ethmoid bone. Due to the good results in this case we prefer endonasal, endoscopic surgery to other surgical procedures described for tumor debulking, e.g. enucleation, cryosurgery or curretages.^5^, ^9^ In our opinion, postoperative follow up should include annual MRI in order to observe tumor size and to evaluate the necessity of repeated surgery which was not necessary in this case 2 years after ESS.

## Conclusion

Although ABCs rarely affect sinuses and/or skull base it should be considered as differential diagnosis of bony lesions of the skull. With clinical, histologic and radiologic examinations ABCs can be diagnosed. Minimal invasive ESS may be used for tumor debulking as symptomatic treatment in radically non resectable lesions.

## Conflicts of interest

The authors declare no conflicts of interest.
